# Integrin-Linked Kinase Links Integrin Activation to Invadopodia Function and Invasion via the p(T567)-Ezrin/NHERF1/NHE1 Pathway

**DOI:** 10.3390/ijms22042162

**Published:** 2021-02-22

**Authors:** Maria Raffaella Greco, Loredana Moro, Stefania Forciniti, Khalid Alfarouk, Stefania Cannone, Rosa Angela Cardone, Stephan Joel Reshkin

**Affiliations:** 1 Department of Bioscience, Biotechnology and Biopharmaceutics, University of Bari, 70126 Bari, Italy; grecorafaella1975@gmail.com (M.R.G.); stefaniafor5@gmail.com (S.F.); stefaniacannone92@gmail.com (S.C.); rosaangela.cardone@uniba.it (R.A.C.); 2 Department of Biomedical Sciences and Human Oncology, University of Bari, 70126 Bari, Italy; 3 Institute of Biomembranes, Bioenergetics, National Research Council, 70126 Bari, Italy; l.moro@ibiom.cnr.it; 4 Alfarouk Biomedical Research Limited Liability Company, Temple Terrace, FL 33617, USA; Alfarouk@hala-alfarouk.org; 5 Zamzam University College, Khartoum 11111, Sudan

**Keywords:** breast cancer, prostate cancer, invasion, invadopodia, pH, integrin signaling

## Abstract

Tumor cell invasion depends largely on degradation of the extracellular matrix (ECM) by protease-rich structures called invadopodia, whose formation and activity requires the convergence of signaling pathways engaged in cell adhesion, actin assembly, membrane regulation and ECM proteolysis. It is known that β1-integrin stimulates invadopodia function through an invadopodial p(T567)-ezrin/NHERF1/NHE1 signal complex that regulates NHE1-driven invadopodia proteolytic activity and invasion. However, the link between β1-integrin and this signaling complex is unknown. In this study, in metastatic breast (MDA-MB-231) and prostate (PC-3) cancer cells, we report that integrin-linked kinase (ILK) integrates β1-integrin with this signaling complex to regulate invadopodia activity and invasion. Proximity ligation assay experiments demonstrate that, in invadopodia, ILK associates with β1-integrin, NHE1 and the scaffold proteins p(T567)-ezrin and NHERF1. Activation of β1-integrin increased both invasion and invadopodia activity, which were specifically blocked by inhibition of either NHE1 or ILK. We conclude that ILK integrates β1-integrin with the ECM proteolytic/invasion signal module to induce NHE1-driven invadopodial ECM proteolysis and cell invasion.

## 1. Introduction

Dissemination of metastatic cells to distant sites is the leading cause of cancer fatality, underlying the need for new therapeutic approaches specifically focusing on invasive tumor cell spreading [1]. However, our limited understanding of invasion has impeded the development of anti-metastatic therapies [2,3,4,5,6]. Successful invasion processes require changes in tumor cell adhesion properties, cell motility and proteolytic remodeling of the extracellular matrix (ECM). It is now well-established that metastatic cells have plasma membrane structures dedicated to driving their increased invasion, called invadopodia. Invadopodia drive cancer cell dissemination through localized proteolytic degradation of the ECM [7,8,9,10,11,12,13,14,15], which has been shown to drive invasion and metastasis, making the understanding of their dynamics crucial to the design of efficient treatments against metastasis [6,16,17,18,19].

Integrins are the main cell adhesion receptors for ECM components. They are heterodimeric transmembrane receptors composed of an α and a β subunit. Integrin engagement with a substrate may trigger the recruitment of specific signaling, scaffolding and cytoskelatal proteins, thereby promoting cancer cell invasion and metastasis [15,20,21]. In cancer cells, activation of β1-integrin increases invadopodia-driven ECM degradation [20,21], and recent work has made it clear that β1-integrin is required for invadopodium stability through adhesion to the ECM and activation of actin polymerization [15,22,23]. β1-integrin initiates invadopodia, promotes their maturation by interacting with the tyrosine kinase, Arg, to phosphorylate cortactin [23,24] and directly recruits the EGFR to a shared lipid raft complex in the invadopodia membrane, where it is restrained by binding the phosphorylated form of the scaffolding protein NHERF1 [25,26]. Additionally, β1-integrin adhesion to the ECM also promotes active, invadopodia focal ECM proteolysis through the phosphorylation of ezrin at T567 [25]. This results in the formation of a “protein–protein” signal complex dedicated to the regulation of invadopodial proteolytic function and subsequent invasive and metastatic potential: the β1-integrin/p-ezrin/NHE1/p-NHERF1 “invadosome” localized in invadopodia that regulates their Na^+^/H^+^ exchanger type 1 (NHE1)-driven proteolytic activity [25,27,28].

It is now well-known that the NHE1 is localized in invadopodia and drives β1-integrin-stimulated invadopodia formation and proteolytic activity, firstly, through the acidification of the extracellular nanospace around invadopodia which drives ECM proteolysis [29] and, secondly, through the alkalinization of the invadopodial cytosol which causes the release of cofilin from cortactin to stimulate the dynamic process of invadopodia protrusion [30,31]. Invadopodia-dependent invasion is activated by both EGF [29,31,32,33] and tumor hypoxia [34] through their stimulation of invadopodial NHE1. However, how β1 signaling is connected to NHE1-driven invadopodia activity promoting tumor invasion is still not completely understood, making the elucidation of the basic mechanisms of invadopodia-driven tumor invasion a major challenge in tumor biology [7,9,10,35].

A candidate could be the scaffolding protein, integrin linked kinase (ILK), a kinase that, by directly interacting with the β1-integrin cytoplasmic domain, links β1-integrin to down-stream signal systems. ILK is over-expressed in aggressive human tumors, promotes cellular transformation, cell survival, epithelial mesenchymal transition (EMT) and metastasis and is associated with poor prognosis [15,36,37,38,39,40] (https://www.proteinatlas.org/ENSG00000166333-ILK/; accessed on 28 January 2021). Moreover, a role of ILK in the maturation of invadopodia into degradative protrusions has been demonstrated. Specifically, knocking-down ILK protein levels with small hairpin RNA led to a reduction in membrane type-I matrix metalloproteinase (MMP) trafficking to invadopodia and of their degradation of the ECM [41,42,43]. On the contrary, ILK-knockdown no longer affected invadopodia formation and stability in integrin-inhibited cells, indicating the critical role of ILK in mediating integrin-dependent invadopodia function. However, its specific mechanism(s) of action involved in driving integrin-dependent invadopodia function has not been described.

In this study, we explored the possibility that β1-integrin–ILK signaling may act as a master integrator of the invadopodia protein–protein complex that regulates invadopodia proteolytic function and subsequent invasion through the activity of NHE1. We observed that ILK is expressed in invadopodia of invasive breast and prostate cancer cells where it forms protein–protein complexes with NHE1, β1-integrin, NHERF1 and ezrin phosphorylated at T567. Furthermore, ILK regulated both β1-integrin- and NHE1-driven invadopodial ECM proteolysis and cell invasion, thus promoting an invasive phenotype in breast and prostate cancer cells in vitro by coordinating an ECM proteolytic/invasion signal module.

The present study adds ILK as an essential component in the molecular mechanisms driving cancer cell invasion, making it a potential marker for pre-symptomatic cancers and exploitable as a therapeutic target in those cancers.

## 2. Results

### 2.1. ILK Co-Localizes with β1-Integrin, NHERF1, p-Ezrin and NHE1 at Sites of Focal ECM Proteolysis

We started by analyzing the direct associations between ILK, β1-integrin p-ezrin, NHE1 and NHERF1 at proteolytically active invadopodia, utilizing in situ proximity ligation assay (in situ PLA), which can measure endogenous protein–protein interactions occurring within 40 nm (please see Materials and Methods for further description). Invadopodia ECM proteolysis was visualized using a protocol based on the degradation-dependent release of fluorescence of a quenched fluorophore (DQ Green-BSA) dissolved in Matrigel, where proteolysis of the ECM was measured as the amount of focal fluorescence unquenched by proteolysis [29]. When measured with high-resolution microscopy, this allowed for simultaneous quantification and mapping of focal proteolytic activity, which permitted a more exact co-localization between focal digestion and proteins, or protein complexes, of interest. As an experimental system, we used MDA-MB-231 and PC-3 cancer cells, two cell lines derived from metastatic lesions of breast and prostate carcinoma, respectively, which are considered to be models of advanced, aggressive cancer.

**Figure 1:**
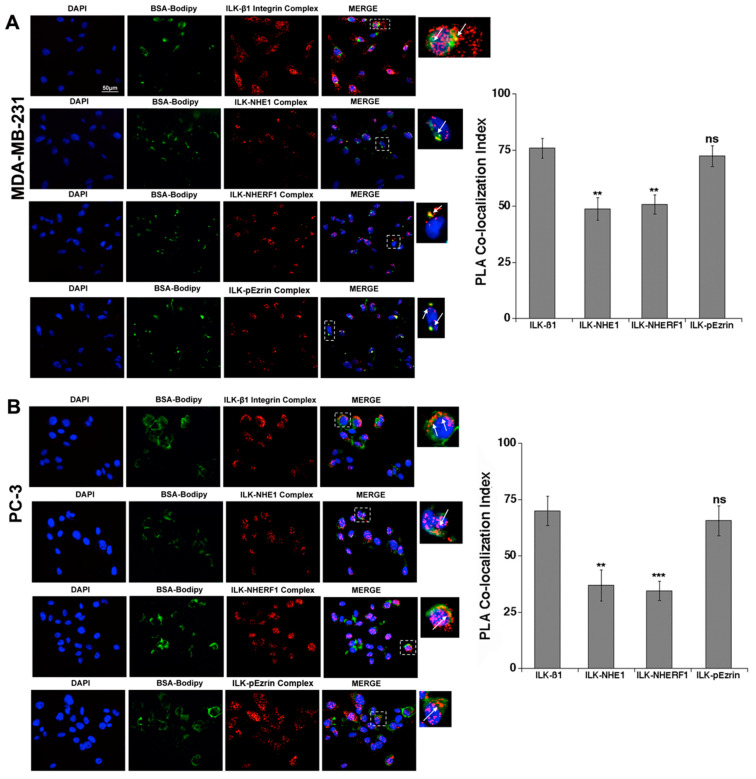
ILK formed protein–protein complexes with β1-integrin receptor, p-ezrin, NHERF1 and NHE1 in areas of focal digestion of Matrigel in MDA-MB-231 (**A**) and PC-3 (**B**) cells. To better visualize invadopodial focal digestion and protein–protein complex localization in Matrigel, we utilized PLA for each protein–protein complex together with in situ zymography using the quenched fluorescent substrate, DQ Green-BSA. Therefore, quantifiable fluorescence was released only upon digestion of the matrix. After the cells digested the fluorogenic substrate (green), the cells were fixed for subsequent PLA analysis (red). The white arrows indicate areas of co-localization of BSA-Bodipy with the PLA signal. The histograms display the analysis of co-localization of ILK with the other proteins (PLA co-localization index) in the specific area of focal proteolysis in ECM digesting cells. Mean ± S.E.M., *n* = 6, ns: non-significant, ** *p* < 0.01, *** *p* < 0.001 for co-localization index compared to the ILK-β1 PLA analysis.

We first examined the co-expression of ILK with each of the above proteins and their association with ECM focal digestion using epifluorescence in cells cultured for 6 h on DQ Green-BSA-Matrigel. As can be seen in Figure 1, ILK co-localized with β1-integrin, NHE1, NHERF1 and p-ezrin, principally in areas of focal ECM proteolysis in both breast MDA-MB-231 (Figure 1A) and prostate PC-3 (Figure 1B) cancer cells. Analysis of the protein–protein co-localization PLA signal (PLA co-localization index) demonstrated that ILK highly co-localized with β1-integrin and p(T567)-ezrin, suggesting that these two protein pairs were very close to each other in the complex and were expressed in all the invadopodia. The co-localization index of the PLA pairs ILK/NHE1 and ILK/NHERF1 were lower in each cell line, suggesting that these two proteins were further away from ILK in the protein–protein complex than were p-ezrin and β1-integrin. Furthermore, Li’s intensity correlation quotient (ICQ) analysis of the images [29] revealed high co-dependence between the distribution of the various PLA complexes and the DQ-BSA proteolysis signal (ICQ = 0.403 ± 0.015; 0.381 ± 0.021; 0.314 ± 0.019 and 0.334 ± 0.015 (*n* = 5 independent fields, *p* < 0.001) for ILK-β1-integrin, ILK-p-ezrin, ILK-NHERF1 and ILK-NHE1, respectively).

**Figure 2:**
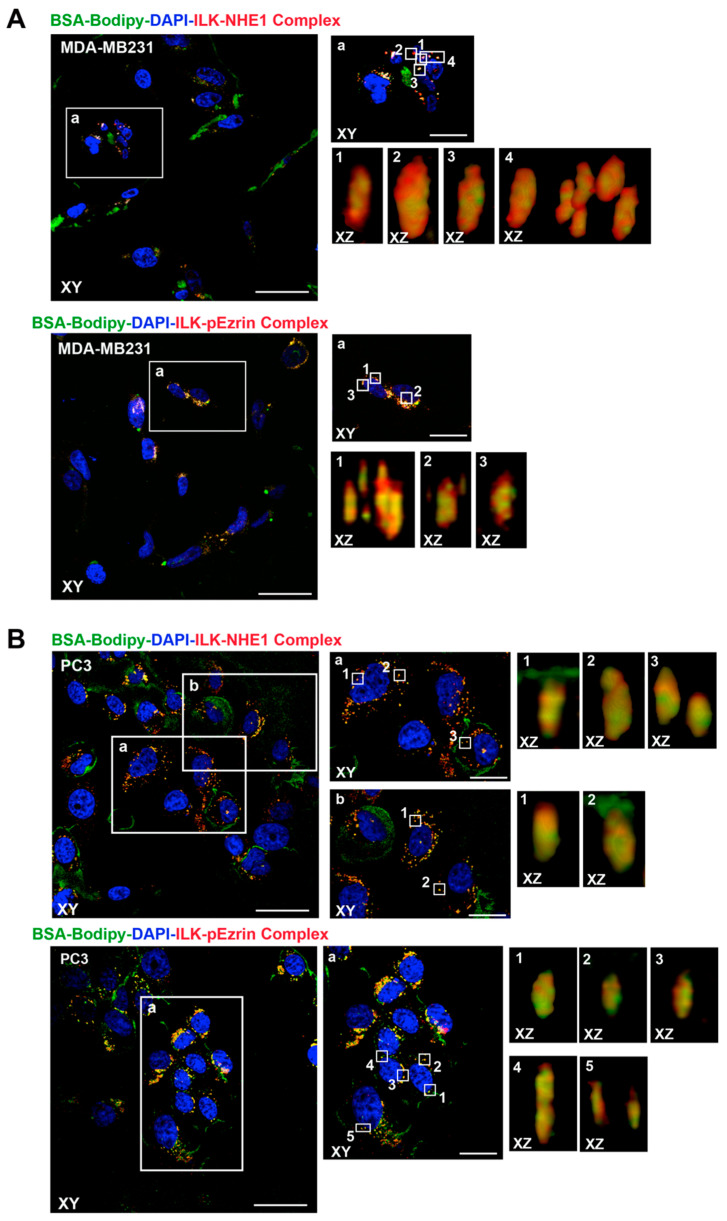
ILK formed protein–protein complexes with NHE1 and p-ezrin within invadopodia in MDA-MB-231 (**A**) and PC-3 (**B**) cells. Cells seeded on Matrigel were allowed to digest the green fluorogenic substrate (DQ) and PLA co-localization assays after fixation. Confocal images in axial planes taken at the bottom of the cells (XY) of a typical region showed protein–protein complexes (red) and digestion (green) localization. In each field, zoomed sections (XZ) reconstructed by alpha blending analysis of the indicated regions of interest (white box) are shown on the right. Importantly, protein–protein complexes (red) and digestion (green) were co-localized in protrusive digestive structures on the ventral cell surface. Scale bars = 10 µm (XY) and 5 µm (XZ).

NHE1 and p(T567)-ezrin play an important role in regulating the function of invadopodia [25,29]. To further explore the structure of these protein–protein signal complexes within invadopodia, we next examined the co-expression of ILK with NHE1 and p(T567)-ezrin using confocal microscopy. This permitted the reconstruction of invadopodia and determination of the co-localization of ILK-NHE1 and ILK-p-ezrin with focal ECM proteolysis at a finer scale. As seen in Figure 2, ILK highly co-localized with both NHE1 and p(T567)-ezrin within invadopodia structures as visualized by the strong co-localization of the complexes with the focal ECM proteolysis of both MDA-MB-231 (Figure 2A) and PC-3 (Figure 2B) cells. Z sectioning and 3D reconstruction (alpha blending), together with RGB analysis, revealed that invadopodia were approximately 1 µm in diameter and 3–5 µm in length and that ILK co-localization with both NHE1 and p(T567)-ezrin occurred within the structures of invadopodial focal digestion.

Altogether, these data suggest that stimulation of cancer cells to develop invadopodia by plating on ECM occurs through the formation of a protein–protein complex formed by β1-integrin, ILK, p(T567)-ezrin, NHERF1 and NHE1.

### 2.2. Role of ILK and NHE1 in Regulating β1-Integrin-Driven Invadopodia Proteolytic Activity

**Figure 3:**
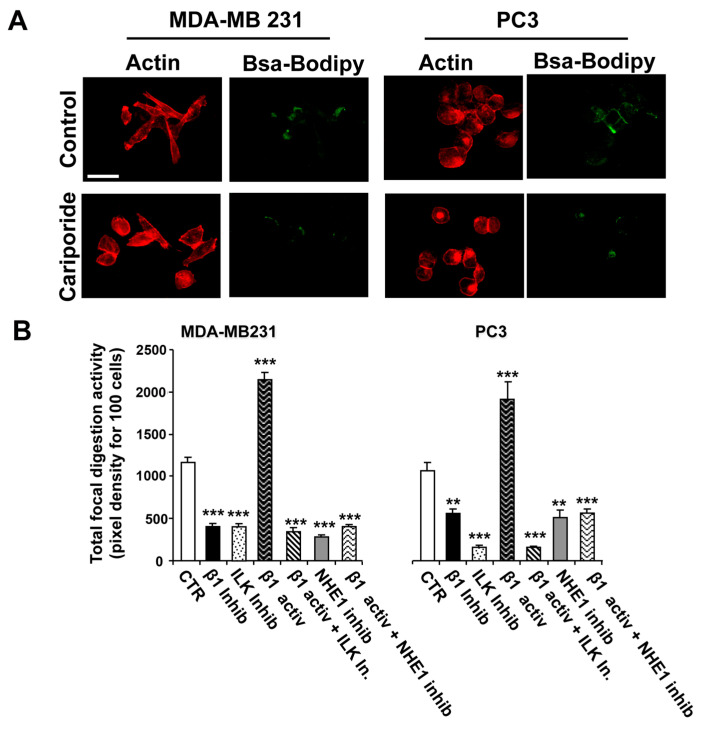
ILK and NHE1 activity are necessary for β1-integrin-driven invadopodia proteolytic activity. To examine the role of NHE1 and ILK in invadopodial-dependent focal digestion of the ECM, MDA-MB-231 and PC3 cells were plated on Matrigel with DQ-Green BSA and, 1 h later, were treated with either the NHE1 inhibitor, ILK inhibitor, β1-integrin inhibiting antibody or β1-integrin activating antibody, with the β1-integrin activating antibody added in either the presence or the absence of the specific NHE1 or ILK inhibitor. After 24 h, ECM digestion was analyzed using fluorescence microscopy for a series of individual cells as described in the Materials and Methods. (**A**) Typical experiment showing control and cariporide-treated cell. (**B**) Histograms showing Mean ± S.E.M., *n* = 4, ** *p* < 0.05, *** *p* < 0.001 for focal proteolysis compared to the control cells.

The results depicted in Figure 2 demonstrated strong co-localization of the β1-integrin-ILK-NHE1 complex with focal ECM proteolysis. To further confirm the regulatory role of this protein–protein complex in cancer cell invasion, we assessed global invadopodia-mediated ECM proteolysis as described in the Materials and Methods. Cells that had been seeded on DQ-labeled Matrigel were treated as follows: 5 mg/mL of either a β1-integrin activating antibody (P4G11) or inhibiting antibody (P5D2) in the absence or presence of 5 µM of the ILK inhibitor, Cpd22, or 5 µM of the specific NHE1 inhibitor, cariporide, with typical experiments for control and cariporide-treated cells shown in Figure 3A.

Analysis of Matrigel proteolysis revealed that both breast (Figure 3B, left panel) and prostate (Figure 3B, right panel) cancer cell lines responded similarly to β1-integrin activation (P4G11 antibody) or inhibition (P5D2 antibody) alone and in combination with the inhibition of ILK (Cpd22, 5 µM) or NHE1 (cariporide, 5 µM). Inhibition of β1-integrin reduced focal invadopodia proteolytic activity by about 65% in MDA-MB-231 and 55% in PC-3 cells, while inhibition of ILK alone reduced this proteolysis by about 70% in both cell lines. Stimulation of β1-integrin increased invadopodia proteolytic activity almost twofold in both cell lines, and the simultaneous inhibition of either ILK or NHE1 together with β1-integrin stimulation blocked this increased activity to levels similar to ILK or NHE1 inhibitors alone.

### 2.3. ILK and NHE1 Mediate β1-Integrin-Driven Invasion

**Figure 4:**
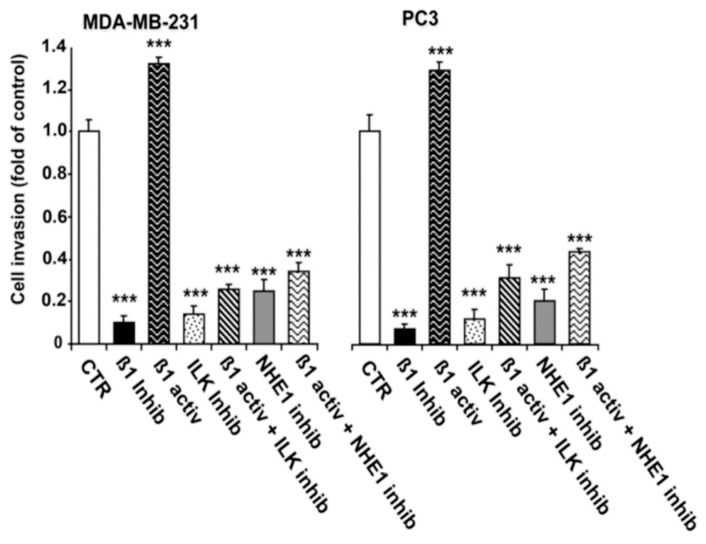
ILK and NHE1 activity are necessary for β1-integrin-driven invasion. To examine the roles of ILK and NHE1 in an invasive capacity, MDA-MB-231 and PC3 cells were stimulated with the β1 integrin activating antibody and treated with either the ILK inhibitor or the β1 integrin inhibiting antibody in the presence or absence of the specific NHE1 inhibitor, cariporide. Cell invasion was analyzed quantitatively by fluorescent labeling of cells that had traversed 8 µm polycarbonate membranes coated with 5 mg/mL Matrigel (Chemicon Int., Livermore, CA, USA) as described in the Materials and Methods. Mean ± S.E.M., *n* = 4, *** *p* < 0.001 compared to control cells.

Considering the above role of β1, ILK and NHE1 in regulating invadopodia-mediated ECM proteolysis, we next tested their role in invasion using a 3D invasion assay where the cells had to cross a thick layer of Matrigel. As can be seen in Figure 4, in invasion, both breast (left panel) and prostate (right panel) cell lines also responded similarly to β1-integrin activation (P4G11 antibody) or inhibition (P5D2 antibody) alone and in combination with the inhibition of ILK (Cpd22, 5 µM) or of NHE1 (cariporide, 5 µM). Inhibition of β1-integrin reduced invasion levels by about 0.65% in MDA-MB-231 and 55% in PC-3 cells, while inhibition of ILK alone reduced invasion by about 70% in both cell lines. Stimulation of β1-integrin increased invasive activity by about 40% in both cell lines, and the simultaneous inhibition of either ILK or NHE1 together with β1-integrin stimulation blocked this increased invasion to levels only slightly higher than that of the ILK or NHE1 inhibitors alone.

**Figure 5:**
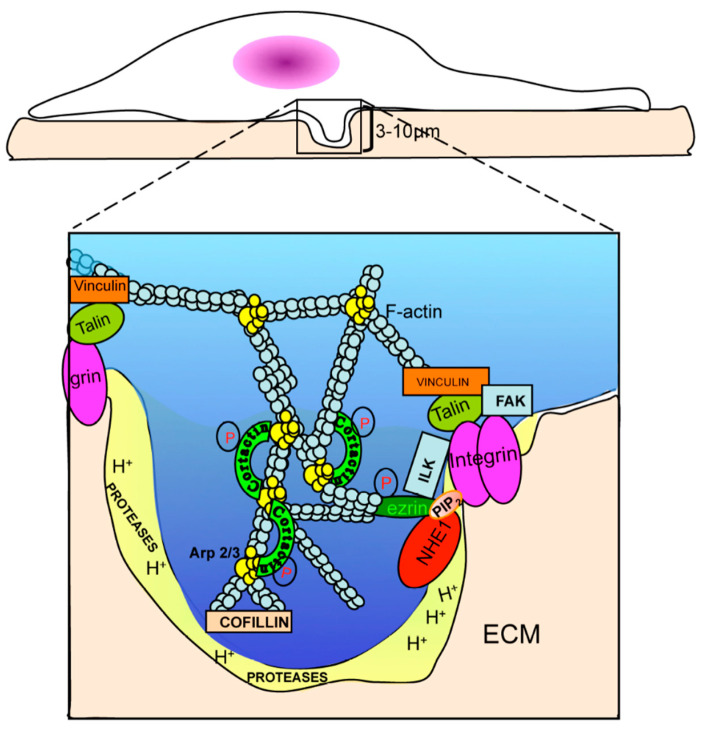
Model of the localization and role of ILK in NHE1-driven invadopodia formation and function. The insert is a magnification of the cellular membrane extrusion, the invadopodia, into the ECM. Invadopodia are F-actin-enriched membrane protrusions responsible for ECM degradation, whose formation is activated by β1-integrin binding to the ECM. This results in β1-integrin binding to and its activation of ILK and in the phosphorylation of the adapter protein, ezrin, at threonine 567. P-ezrin binds to NHE1 and the cytoskeleton and shifts the complex to PIP_2_-rich lipid rafts where NHE1 is activated [25]. NHE1, with its two functions as a scaffolding protein and ion exchanger, leads to membrane protrusion and proteolysis. As a proton transporter, NHE1 promotes invasion through its control of the acidification of the peri-invadopodial space, where NHE1 proton-secreting activity and proteases act in concert to degrade the ECM during invasion. The proteases cathepsin B, D and L, urokinase plasmogen activator and the matrix metalloproteinases MMP-2 and MMP-9 are released extracellularly, while MT1-MMP is associated with the membrane and participates, together with cathepsin B, in the processing of inactive pro-MMP-2 into active MMP-2. Glycolytic enzymes are enriched in invadopodia, leading to the localized production of intracellular protons secreted via active NHE1, resulting in peri-invadopodial acidification favorable for the activity of the various proteases localized in this sub-cellular region. Furthermore, the NHE1-dependent alkalinization of the invadopodia cytosol results in phosphorylation of cortactin with subsequent release of cofilin, which promotes actin polymerization, growth of the invadopodia cytoskeleton and invadopodia protrusion. NHE1 also promotes invadopodial formation via its interaction with the cytoskeleton through binding to the actin-anchoring protein, phospho-ezrin.

Altogether, these data suggest that cancer cells develop functional invadopodia and perform subsequent invasion by establishing a compartmentalized, functional “signalsome” inside invadopodia, composed of β1-integrin, ILK, NHE1, p-ezrin and p-NHERF1. These data demonstrate that ILK functionally participates in the well-known β1-integrin/NHE1 protein complex regulation of invadopodia activity. Figure 5 shows a model of the possible structure of this invadopodial protein–protein signalsome.

## 3. Discussion

One of the primary spatial cues leading to the formation of invadopodia, degradation of the underlying ECM via focal proteolysis and permission of tumor cell invasion is the activation of β1-integrin through cell-substrate adhesion [7,8,9,10,11,12,13,14]. We have previously identified a signaling complex that links the binding of β1-integrin to the ECM to the assembly of a p(T567)-ezrin/p-NHERF1 signal protein complex, which activates NHE1 and subsequent invadopodia formation and proteolytic activity [25,28,29,33,44]. However, the structural mechanisms by which β1-integrin and this signalsome are functionally interconnected in invadopodia have only started to be defined.

While in vitro studies have demonstrated that ILK increases invasion and metastasis (see Introduction), the precise mechanism(s) involved in its role in driving metastasis are unknown. Therefore, we determined ILK expression in invadopodia, its association with other invadopodial proteins and its role in mediating both invadopodia proteolytic function and invasion. In particular, we aimed to elucidate the role of ILK in the invadopodial β1-integrin receptor-promoted p(T567)-ezrin/p-NHERF1/NHE1 complex in driving invadopodial proteolysis and invasion.

In this study, we demonstrated that through binding to the ECM, the β1-integrin receptor promotes its association with ILK, facilitating the formation of a protein–protein β1-integrin/p(T567)-ezrin/NHERF1/NHE1 signaling complex that promotes NHE1-dependent invadopodia formation and proteolytic activity [11,29,31]. This preferential invadopodia co-localization of ILK with β1-integrin, its adapter proteins (p-ezrin and NHERF1) and the integrator/driver protein, NHE1, was firstly identified by combining PLA measurements of ILK associated with the different signalsome components with the in situ assay for invadopodial focal ECM proteolysis. We found that, within the areas of focal invadopodial ECM proteolysis, ILK most stringently associated with the β1-integrin and p(T567)-ezrin components of the complex and somewhat less stringently with NHERF1 and NHE1 to finely regulate the development and function of invadopodia. These data were then confirmed by confocal microscopy combined with PLA, in which we observed the localization of PLA protein–protein pairs within the reconstructed invadopodial complex (Figure 2).

Lastly, we determined the role of ILK in this signaling cassette in regulating invadopodia proteolytic action (Figure 3) and invasive capacity (Figure 4) by using inhibitors of both ILK (Cpd22) and NHE1 (cariporide) together with either an activator (P4G11) or an inhibitor (P5D2) of β1-integrin. We found that specific activation of β1-integrin with an activating antibody (P4G11) stimulated both invadopodia-dependent focal proteolysis (Figure 3) and invasion (Figure 4) that were abrogated by the pharmacological inhibition of both ILK and NHE1. The hypothesis that invasion is driven in breast and prostate cancer cells, at least in part, through the invadopodia was further supported by the similar regulatory pattern of ECM proteolysis with invasion. The human cell lines used in this study were either p53-null (PC-3) or contained a mutant p53 (MDA-MB-231). Future experiments performed in invasive cancer cells containing wild-type p53 will allow for the assessment of whether loss of p53 plays a role in the structural and functional definition of the β1-integrin-dependent signalsome in invadopodia. Because this study was conducted in 2D, it does not provide details on whether the invadopodial β1-integrin/ILK/NHE1/p(T567)-ezrin/NHERF1 signalsome is constitutively present and/or equally functional during essential steps of the metastatic cascade, i.e., cancer cell local invasion, intravasation and extravasation. Future studies performed in animal models will establish the in vivo significance of the invadopodial signalsome during metastatic dissemination.

Altogether, these data demonstrate that ILK functionally participates in the formation of a compartmentalized functional “signalsome” composed of β1-integrin, NHE1, p(T567)-ezrin and NHERF1 that drives invadopodial proteolytic activity and subsequent invasion. We believe that ILK is a potential marker for the detection and identification of pre-metastatic cancers and, therefore, could be exploited as an anti-metastasic target in these cancers. A model of the possible structure of this invadopodial protein–protein signalsome is presented in Figure 5 and the Graphical Abstract.

## 4. Materials and Methods

### 4.1. Cell Culture and Transfection of Constructs

MDA-MB-231 cells [45] and PC-3 cells [46] were cultured as previously described. The ILK inhibitor, Cpd22, was purchased from EMD Millipore (Burlington, MA, USA). The NHE1 inhibitor (cariporide) and β1-integrin activating (P4G11) and inhibitory (P5D2) antibodies were purchased from Santa Cruz Biotechnology, (Santa Cruz, CA, USA).

### 4.2. Matrigel Layer Preparation and Invadopodia Activity Assay Using In Situ Zymography

Experiments were conducted in 4 mg/mL Matrigel containing a quenched BODIPY linked to BSA (DQ-Green BSA) as previously described [29]. Focal proteolysis produced fluorescence on a black background which was used both to quantify proteolytic activity levels and in co-localization analysis. The quantity of invadopodia activity was determined with the following measurements: (i) percent of cells with active invadopodia, (ii) number of invadopodia per active cell and (iii) pixel density of digestion performed by individual invadopodia. The mean total actual invadopodia proteolytic activity for 100 cells was then calculated as follows: invadopodial index = percentage of invadopodia-positive cells (proteolytically active areas also positive for both actin/cortactin) × mean pixel density of invadopodia/cell.

### 4.3. Invasion across Matrigel Layer in Boyden Chambers

A quantitative measure of in vitro invasion was assayed by cells traversing an 8 µm polycarbonate membrane coated with 5 mg/mL Matrigel (Chemicon Int., Livermore, CA, USA) as previously described [47]. Cell fluorescence was read using a Cary Eclipse fluorescence plate reader (Varian) at 480/520 nm.

### 4.4. Proximity Ligation Assay

To analyze the potential direct association between ILK, β1-integrin, NHE1, p(T567)-ezrin and NHERF1, we used an in situ proximity ligation assay (in situ PLA) (Duolink II Kit; Sigma-Aldrich, Arklow, Ireland), which can detect endogenous protein–protein interactions that occur within 40 nm [48] and provides a fluorescent signal (red) only when the two target proteins are co-localized. The advantages of PLA are that it has greatly improved sensitivity for establishing endogenous protein–protein interactions and gives in situ information about whether these co-localizations occur in specific intracellular compartments. PLA complexes were detected either with a Nikon TE 2000S epifluorescence microscope, equipped with a MicroMax 512BFT CCD camera (Princeton Instruments, Trenton, NJ, USA) using a Nikon lamp shutter with a mercury short-arc photo-optic HBO 103 W/2 lamp for excitation (OSRAM GmbH, Augsburg, Germany) or, at 600× magnification in oil immersion, with a laser scanning confocal microscope (LSCM) (C1/TE2000-U; Nikon Instruments SpA, Sesto Fiorentino, FI, Italy), equipped with He/Ne 633 and Argon 488 lasers with 495–519 (B2-A) and 642–660 (Cy5) nm excitation filters. All images were taken under Plan Apo 60XA/1.40 NA oil objective (Nikon, Japan), and scanning was conducted with 25–30 optical series from the top to the bottom of the cell with a 0.45 µm step size. Parameters related to fluorescence intensity were maintained at constant values in all measurements. The tighter the association of the two proteins, the higher the number of positive points per cell and the percentage of cells positive for the signal. Therefore, mean pixel density for each cell was the sum of all points contained in that cell. The quantity of co-localization was determined with the following measurements: (i) pixel density of co-localization and (ii) percent of cells with co-localization. From these measures, the mean total co-localization for 100 cells was calculated as follows: co-localization index = percentage of co-localization-positive cells × mean pixel density of co-localized points/cell. This analysis was combined with the Matrigel degradation assay described above to measure invadopodia-driven ECM digestion. To determine the potential direct association between ILK, β1-integrin, NHE1, p(T567)-ezrin and NHERF1 at proteolytically active invadopodia, we analyzed the overlap of PLA signal and proteolysis signal with Li’s intensity correlation quotient (ICQ) analysis [29] of the images in Figure 1 and the 3D reconstruction of the invadopodia in confocal microscopy (Figure 2). The ICQ of cells in five independent fields for each cell type and treatment was calculated using the JACoP image analysis package plugin in ImageJ. ICQ values were near zero when apparent co-localization was due to random staining or very high intensities in one window, while if the two intensities were interdependent (co-localized), the values were positive with a maximum of 0.5. In addition to being useful for identifying potential low-affinity interactions within protein complexes that may be missed in high-affinity co-immunoprecipitation or pull-down experiments, ICQ analysis provides information on cellular localization of the signals.

### 4.5. Image Analysis

For every image, a Z-stack was acquired using the Metamorph software (Universal Imaging Corp, West Chester, PA, USA), and every three-color stack (red, green and blue) was the sum of the three stacks (one for each color) acquired separately in black and white (B/W). Before image analysis, each stack was deconvolved using the AutoDeblur 9.1 function of the AutoQuant software (Troy, NY, USA) and then merged by transforming the three channels corresponding to red, green and blue into a single two-color stack using the ‘RGB merge’ command of the ImageJ software. To verify co-localization, the three separate B/W stacks were analyzed with the “co-localization” plugin of ImageJ with a ratio of 97 and threshold of 50 for both channel 1 and 2. Then, selecting the “co-localized points (8 bit)” option, a new stack was obtained where the co-localized pixels appeared white on a black background, which was then converted into a voxel-gradient (VG) using the shading function of AutoVisualize (AutoQuant software, Troy, NY, USA) for observation of the 3D co-localization zones in a volume.

The random or co-dependent nature of the above calculated “apparent” dye-overlap co-localizations was tested using Li’s intensity correlation quotient (ICQ) as described above.

### 4.6. Statistical Procedures

In the in vitro experiments, an unpaired Student’s *t*-test was applied to analyze the statistical significance between treatments, in which *p* < 0.05 was considered significant. All comparisons were performed with InStat (GraphPad Software, San Diego, CA, USA).

## Data Availability

Data is contained within the article.
